# Supplementary material for: Effects of breed, management and personality on cortisol reactivity in sport horses
Source: PLoS One. 2019 Dec 2;14(12):e0221794. doi: 10.1371/journal.pone.0221794 (PMC6886778; doi:10.1371/journal.pone.0221794)
Supplement: S1 File — The questionnaire consists of two parts. The first part contains questions about demographic and management factors. The second part about personality traits was adapted from a previously validated questionnaire by Momozawa et al. [45]. In addition to the English version, the German and French translations of the questionnaire are included, as they were used in the study. (PDF) [file pone.0221794.s001.pdf]

Questionnaire (English)

Part 1:

Date: ______________

General questions:

UELN/SVPS Number: ____

Name of the horse: ____

Horse details (Breed, gender, date birth, color): _____________________________________

Discipline (f.e. show jumping, dressage etc.): _____________________________________

Name owner and rider: ____

Phone number of responsible person: ____

Email of responsible person: ____

Location of the horse (Canton): ____

Origin/Ancestry: F: _____________________MF: __________________________________

For how long do you own/know the horse? _______________________________________

Temperament of the horse?

☐ calm ☐ temperamental ☐ nervous

Husbandry

1) Does the horse live in a group with other horses?

☐ yes ☐ only on pasture

☐ no

2) How many hours does the horse spent outside of its stall (riding, pasture etc.)?

☐ 0-1h ☐ 1-4h ☐ 4-12h ☐ > 12h

Feeding:

3) During feeding, does the horse show aggressiveness against other horses or humans?

☐ yes ☐ no

4) How is the horse's appetite?

☐ good, eats everything immediately

☐ moderate, eats with breaks and sometimes it does not eat all roughage

☐ poor, eats only slowly and not everything, only favorite food

Training:

5) Number of training days per week?

☐ < 1 times ☐ 4-5 times ☐ 7 times
☐ 1-3 times ☐ 6 times

6) How many different people ride/train the horse?

☐ 1 ☐ 2 ☐ 3 ☐ >3

7) How many times per year does the horse go to competitions?

☐ 1-3 times ☐ 4-8 times ☐ 9-15 times ☐ 16-25 times ☐ more

8) What is the horse's level of performance at horse shows? ________________

9) How is the horse's performance?

☐ very good/good
☐ moderate
☐ poor

10) How is the horse's motivation?

☐ very good/good
☐ moderate
☐ poor

11) Does the horse's motivation change during work (several answers possible)?

☐ no

If yes:
☐ in the beginning not good
☐ in the middle not good
☐ in the end not good
☐ always not good
☐ always different
☐ during competition season not good

12) Does the horse enjoy moving around (riding, pasture etc.)?

☐ very much
☐ enough
☐ yes during riding, not at pasture
☐ yes at pasture, not during riding
☐ no

Stereotypies:

- weaving?	☐ never	☐ sometimes	☐ regularly	☐ excessive
- crib-biting (grasping fixed object)?	☐ never	☐ sometimes	☐ regularly	☐ excessive
- crib-biting (windsucking)?	☐ never	☐ sometimes	☐ regularly	☐ excessive
- stall walking?	☐ never	☐ sometimes	☐ regularly	☐ excessive
- teeth sharpening?	☐ never	☐ sometimes	☐ regularly	☐ excessive

Behavioral problems:

- dependency on other horses?	☐ never	☐ sometimes	☐ regularly	☐ excessive
- biting?	☐ never	☐ sometimes	☐ regularly	☐ excessive
- kicking?	☐ never	☐ sometimes	☐ regularly	☐ excessive
- bucking?	☐ never	☐ sometimes	☐ regularly	☐ excessive
- rearing up?	☐ never	☐ sometimes	☐ regularly	☐ excessive
- kicking against the wall?	☐ never	☐ sometimes	☐ regularly	☐ excessive

never	= no signs of stereotypies/behavioral problems
sometimes	= stereotypies/behavioral problems appear not every day
regulary	= stereotypies/behavioral problems appear every day in relation to a certain stimulus (f.e. after feeding, after riding etc.)
excessive	= stereotypies/behavioral problems appear every day, except during riding etc.

Clinical signs:

Did you notice one or several of the following clinical signs:

☐ yawning

☐ weight loss

☐ problems gaining weight

☐ teeth grinding

Part 2:

(Tick the correct number on the scale)

1) (Nervousness) Becomes nervous about insects, noises etc.:

Nervous Calm

1 –---- 2 –---- 3 -----– 4 ------ 5 –---- 6 –---- 7 –---- 8 ------ 9

2) (Concentration) Easy trainable and not distracted by the environment:

poor excellent

1 –---- 2 –---- 3 -----– 4 ------ 5 –---- 6 –---- 7 –---- 8 ------ 9

3) (Self-reliance) Is at ease, if left alone, away from the herd:

restless at ease

1 –---- 2 –---- 3 -----– 4 ------ 5 –---- 6 –---- 7 –---- 8 ------ 9

4) (Trainability) Easy and promptly trainable:

poor excellent

1 –---- 2 –---- 3 -----– 4 ------ 5 –---- 6 –---- 7 –---- 8 ------ 9

5) (Excitability) Easily getting excited:

excitable not excitable

1 –---- 2 –---- 3 -----– 4 ------ 5 –---- 6 –---- 7 –---- 8 ------ 9

6) (Friendliness people) Is never aggressive or fearful:

unfriendly friendly

1 –---- 2 –---- 3 -----– 4 ------ 5 –---- 6 –---- 7 –---- 8 ------ 9

7) (Curiosity) Is interested in novel objects and approaches them:

rarely frequently

1 –---- 2 –---- 3 -----– 4 ------ 5 –---- 6 –---- 7 –---- 8 ------ 9

8) (Memory) Memorizes what it learned or what was trained:

poor excellent

1 –---- 2 –---- 3 -----– 4 ------ 5 –---- 6 –---- 7 –---- 8 ------ 9

9) (Panic) Gets excited to an abnormal extent:

frequently never

1 –---- 2 –---- 3 -----– 4 ------ 5 –---- 6 –---- 7 –---- 8 ------ 9

10) (Cooperation) Is cooperative with caretakers when handled:

never frequently

1 –---- 2 –---- 3 -----– 4 ------ 5 –---- 6 –---- 7 –---- 8 ------ 9

11) (Inconsistent emotionality) Is unpredictable from day to day:

inconsistent consistent

1 –---- 2 –---- 3 -----– 4 ------ 5 –---- 6 –---- 7 –---- 8 ------ 9

12) (Stubbornness) Is obstinate once it resists a command:

stubborn obedient

1 –---- 2 –---- 3 -----– 4 ------ 5 –---- 6 –---- 7 –---- 8 ------ 9

13) (Docility) Is docile in general:

active docile

1 –---- 2 –---- 3 -----– 4 ------ 5 –---- 6 –---- 7 –---- 8 ------ 9

14) (Vigilance) Is vigilant about surroundings:

never always

1 –---- 2 –---- 3 -----– 4 ------ 5 –---- 6 –---- 7 –---- 8 ------ 9

15) (Perseverance) Is patient with various stimuli:

impatient patient

1 –---- 2 –---- 3 -----– 4 ------ 5 –---- 6 –---- 7 –---- 8 ------ 9

16) (Friendliness horses) Interacts with other horses in a friendly manner:

unfriendly friendly

1 –---- 2 –---- 3 -----– 4 ------ 5 –---- 6 –---- 7 –---- 8 ------ 9

17) (Competitiveness) Is dominant in antagonistic encounters with other horses:

subordinate dominant

1 –---- 2 –---- 3 -----– 4 ------ 5 –---- 6 –---- 7 –---- 8 ------ 9

18) (Skittishness) Gets surprised easily:

skittish not skittish

1 –---- 2 –---- 3 -----– 4 ------ 5 –---- 6 –---- 7 –---- 8 ------ 9

19) (Timidity) Is timid in a novel environment:

timid audacious

1 –---- 2 –---- 3 -----– 4 ------ 5 –---- 6 –---- 7 –---- 8 ------ 9

20) (Cooperation) Is cooperative during riding:

never always

1 –---- 2 –---- 3 -----– 4 ------ 5 –---- 6 –---- 7 –---- 8 ------ 9

21) (Cooperation) Is cooperative during trailer loading:

never always

1 –---- 2 –---- 3 -----– 4 ------ 5 –---- 6 –---- 7 –---- 8 ------ 9

22) (Cooperation) Is cooperative during transportation?

never always

1 –---- 2 –---- 3 -----– 4 ------ 5 –---- 6 –---- 7 –---- 8 ------ 9

23) (Capacity recovery time) Recovers slowly after putatively stressful events such as competitions or transports:

slowly fast

1 –---- 2 –---- 3 -----– 4 ------ 5 –---- 6 –---- 7 –---- 8 ------ 9

24) (Capacity infections) Is susceptible to infections (e.g. respiratory infections) during or after putatively stressful events such as competitions or transports?

susceptible   not susceptible

1 –---- 2 –---- 3 ----– 4 ------ 5 –---- 6 –---- 7 –---- 8 ------ 9

25) (Capacity inappetence) Shows inappetence during or after putatively stressful events such as competitions or transports?

always   never

1 –---- 2 –---- 3 ----– 4 ------ 5 –---- 6 –---- 7 –---- 8 ------ 9

26) (Capacity discomfort) Shows signs of discomfort during or after putatively stressful events such as competitions or transports (Flehmen, colic signs, increased phases of laying down…)?

always   never

1 –---- 2 –---- 3 ----– 4 ------ 5 –---- 6 –---- 7 –---- 8 ------ 9

Fragebogen (German)

Teil 1:

Datum: ___________________

Allgemeine Fragen:

UELN/SVPS Nummer: ____

Name des Pferdes: ____

Signalement (Rasse, Geschlecht, Geburtsdatum, Farbe): _____________________________

Disziplin (z.B. Springen, Dressur etc.): ____________ __________________________

Name Besitzer/in und Reiter/in: ____

Telefon der zuständigen Person: ____

Email der zuständigen Person: ____

Standort des Pferdes (Kanton): ____

Herkunft/Abstammung: V: ______________________**MV:** ____________________________

Seit wann ist das Pferd in ihrem Besitz/kennen Sie es? ______________________________

Temperament des Pferdes?

☐ gelassen, ruhig ☐ temperamentvoll, aber kooperativ ☐ schnell nervös, eher unkooperativ

Haltung

1) Lebt Ihr Pferd in einer Gruppe?

☐ Ja ☐ nur auf der Weide

☐ Nein

2) Wie viele Stunden verbringt Ihr Pferd durchschnittlich/Tag draussen (Reiten, Weide etc.)?

☐ 0-1h ☐ 1-4h ☐ 4-12h ☐ > 12h

Fütterung:

3) Ist Ihr Pferd futterneidisch?

☐ Ja ☐ Nein

4) Wie ist der Appetit Ihres Pferdes?

☐ gut, frisst sofort alles auf

☐ mässig, frisst mit Pausen und lässt Raufutter manchmal liegen

☐ eher ungenügend, frisst nur langsam und nicht alles, nur „Lieblingsfutter“

Training:

5) Wie oft pro Woche wird das Pferd trainiert?

☐ < 1Mal ☐ 4-5 Mal ☐ 7 Mal

☐ 1-3 Mal ☐ 6 Mal

6) Wieviele verschiedene Leute reiten/trainieren das Pferd?

☐ 1 ☐ 2 ☐ 3 ☐ >3

7) Wie oft pro Jahr startet es an Turnieren?

☐ 1-3 Mal ☐ 4-8 Mal ☐ 9-15 Mal ☐ 16-25 Mal ☐ mehr

8) Auf welchem Niveau startet das Pferd? ________________

9) Bringt das Pferd dem Ausbildungsstand entsprechend gute Leistung?

☐ sehr gut/gut

☐ genügend

☐ ungenügend/schlecht

10) Ist das Pferd motiviert bei der Arbeit?

☐ sehr gut/gut

☐ genügend

☐ ungenügend/schlecht

11) Zeigt das Pferd während der Arbeit Motivationsschwankungen (Mehrfachnennung möglich)?

☐ Nein

Wenn ja:
☐ Anfangs nicht gut
☐ Mitte der Arbeit nicht gut
☐ Ende der Arbeit nicht gut
☐ während jeder Arbeitsphase nicht gut
☐ immer unterschiedlich
☐ während der Saison nicht gut

12) Ist das Pferd bewegungsfreudig (Reiten, Weide etc.)?

☐ Sehr bewegungsfreudig

☐ Genügend bewegungsfreudig

☐ Beim Reiten schon, aber auf der Weide nicht

☐ Auf der Weide schon, aber beim Reiten nicht

☐ Nein, nicht bewegungsfreudig

Stereotypien:

- Weben?	☐ gar nicht	☐ ab und zu	☐ regelmässig	☐ dauernd/exzessiv
- Aufsetzkopper?	☐ gar nicht	☐ ab und zu	☐ regelmässig	☐ dauernd/exzessiv
- Freikopper?	☐ gar nicht	☐ ab und zu	☐ regelmässig	☐ dauernd/exzessiv
- Boxenlaufen?	☐ gar nicht	☐ ab und zu	☐ regelmässig	☐ dauernd/exzessiv
- Zahnwetzen?	☐ gar nicht	☐ ab und zu	☐ regelmässig	☐ dauernd/exzessiv

Verhaltensprobleme.

- Kleben?	☐ gar nicht	☐ ab und zu	☐ regelmässig	☐ dauernd/exzessiv
- Beissen?	☐ gar nicht	☐ ab und zu	☐ regelmässig	☐ dauernd/exzessiv
- Schlagen?	☐ gar nicht	☐ ab und zu	☐ regelmässig	☐ dauernd/exzessiv
- Bocken?	☐ gar nicht	☐ ab und zu	☐ regelmässig	☐ dauernd/exzessiv
- Steigen?	☐ gar nicht	☐ ab und zu	☐ regelmässig	☐ dauernd/exzessiv
- gegen Wand schlagen?	☐ gar nicht	☐ ab und zu	☐ regelmässig	☐ dauernd/exzessiv

gar nicht	= Keine Zeichen von Stereotypien/Verhaltensproblemen
ab und zu	= Stereotypien/Verhaltensprobleme treten nicht täglich auf
regelmässig	= Stereotypien/Verhaltensprobleme treten täglich auf und beziehen sich auf einen bestimmten Stimulus (z. B. nach dem Fressen, nach dem Reiten, etc.)
dauernd/exzessiv	= Stereotypien/Verhaltensprobleme treten den ganzen Tag auf, ausser während dem Reiten, etc.

Symptome:

Ist Ihnen eines oder mehrere der folgenden Symptome bei ihrem Pferd aufgefallen:

☐ Vermehrtes Gähnen

☐ Gewichtsverlust

☐ Probleme an Gewicht zuzunehmen

☐ Zähneknirschen

Teil 2:

(Kreuzen Sie die zutreffende Zahl auf der Skala an)

1) (Nervosität) Wird bei Insekten oder Geräuschen etc. schnell nervös:

Nervös Ruhig

1 –---- 2 –---- 3 -----– 4 ------ 5 –---- 6 –---- 7 –---- 8 ------ 9

2) (Konzentration) Ist gut trainierbar und lässt sich nicht von der Umgebung ablenken.

Schlecht
Ausgezeichnet

1 –---- 2 –---- 3 ----– 4 ------ 5 –---- 6 –---- 7 –---- 8 ------ 9

3) (Selbstvertrauen) Ist ruhig/ausgeglichen, wenn es alleine gelassen wird, weg von anderen Pferden:

Ruhelos
Ausgeglichen

1 –---- 2 –---- 3 ----– 4 ------ 5 –---- 6 –---- 7 –---- 8 ------ 9

4) (Trainierbarkeit) Lässt sich prompt und leicht trainieren:

Schlecht
Ausgezeichnet

1 –---- 2 –---- 3 ----– 4 ------ 5 –---- 6 –---- 7 –---- 8 ------ 9

5) (Erregbarkeit) Ist leicht erregbar:

Leicht erregbar
Nicht erregbar

1 –---- 2 –---- 3 ----– 4 ------ 5 –---- 6 –---- 7 –---- 8 ------ 9

6) (Freundlichkeit gegenüber Menschen) Ist nie aggressiv oder ängstlich:

Unfreundlich
Freundlich

1 –---- 2 –---- 3 ----– 4 ------ 5 –---- 6 –---- 7 –---- 8 ------ 9

7) (Neugier) Ist an neuen Gegenständen interessiert und nähert sich diesen an:

Selten
Häufig

1 –---- 2 –---- 3 ----– 4 ------ 5 –---- 6 –---- 7 –---- 8 ------ 9

8) (Erinnerungsvermögen) Erinnert sich an Gelerntes oder Trainiertes:

Schlecht
Ausgezeichnet

1 –---- 2 –---- 3 ----– 4 ------ 5 –---- 6 –---- 7 –---- 8 ------ 9

9) (Panik) Regt sich übermässig auf:

Häufig
Nie

1 –---- 2 –---- 3 ----– 4 ------ 5 –---- 6 –---- 7 –---- 8 ------ 9

10) (Kooperation) Ist im Umgang/bei der Pflege kooperativ:

Nie
Häufig

1 –---- 2 –---- 3 ----– 4 ------ 5 –---- 6 –---- 7 –---- 8 ------ 9

11) (Launenhaftigkeit) Verhält sich von Tag zu Tag unberechenbar:

Wechselhaft
Gleichbleibend

1 –---- 2 –---- 3 ----– 4 ------ 5 –---- 6 –---- 7 –---- 8 ------ 9

12) (Sturheit) Ist eigensinnig, wenn es sich gegen Hilfen oder Anweisungen wehrt:

Stur
Folgsam

1 –---- 2 –---- 3 ----– 4 ------ 5 –---- 6 –---- 7 –---- 8 ------ 9

13) (Fügsamkeit) Ist im Allgemeinen brav/folgsam:

Aktiv/lebhaft Ruhig/gefügig

1 –---- 2 –---- 3 -----– 4 ------ 5 –---- 6 –---- 7 –---- 8 ------ 9

14) (Wachsamkeit) Ist wachsam gegenüber der Umgebung:

Nie Immer

1 –---- 2 –---- 3 -----– 4 ------ 5 –---- 6 –---- 7 –---- 8 ------ 9

15) (Langmütigkeit) Ist gelassen/unbeeindruckt gegenüber/bei verschiedenen Reizen:

Ungeduldig/Erregbar Langmütig

1 –---- 2 –---- 3 -----– 4 ------ 5 –---- 6 –---- 7 –---- 8 ------ 9

16) (Freundlichkeit mit anderen Pferden) Interagiert mit anderen Pferden auf freundliche Art und Weise:

Unfreundlich Freundlich

1 –---- 2 –---- 3 -----– 4 ------ 5 –---- 6 –---- 7 –---- 8 ------ 9

17) (Konkurrenzverhalten) Ist dominant bei Konflikten mit anderen Pferden:

Untergeordnet Dominant

1 –---- 2 –---- 3 -----– 4 ------ 5 –---- 6 –---- 7 –---- 8 ------ 9

18) (Schreckhaftigkeit) Lässt sich leicht überraschen:

Schreckhaft Nicht schreckhaft

1 –---- 2 –---- 3 -----– 4 ------ 5 –---- 6 –---- 7 –---- 8 ------ 9

19) (Schüchternheit/Ängstlichkeit) Ist schüchtern/zurückhaltend/scheu in einer neuen Umgebung:

Schüchtern Mutig/Kühn

1 –---- 2 –---- 3 -----– 4 ------ 5 –---- 6 –---- 7 –---- 8 ------ 9

20) (Kooperation) Ist beim Reiten kooperativ:

Nie Immer

1 –---- 2 –---- 3 -----– 4 ------ 5 –---- 6 –---- 7 –---- 8 ------ 9

21) (Kooperation) Ist beim Verladen kooperativ:

Nie Immer

1 –---- 2 –---- 3 -----– 4 ------ 5 –---- 6 –---- 7 –---- 8 ------ 9

22) (Kooperation) Ist ruhig während des Transports?

Nie Immer

1 –---- 2 –---- 3 -----– 4 ------ 5 –---- 6 –---- 7 –---- 8 ------ 9

23) (Belastbarkeit) Braucht nach grösseren Anstrengungen wie Transporten und Turnieren lange, um sich zu erholen:

Erholt sich sehr langsam Erholt sich sehr schnell

1 –---- 2 –---- 3 -----– 4 ------ 5 –---- 6 –---- 7 –---- 8 ------ 9

24) (Belastbarkeit) Ist nach grösseren Anstrengungen wie Transporten oder Turnieren anfälliger für Infekte, z.B. der Atemwege (Husten, Nasenausfluss etc.)?

Sehr anfällig Gar nicht anfällig

1 –---- 2 –---- 3 -----– 4 ------ 5 –---- 6 –---- 7 –---- 8 ------ 9

25) (Belastbarkeit) Zeigt während oder nach grösseren Anstrengungen wie Transporten und Turnieren vermehrt Anzeichen von Inappetenz?

Immer Nie

1 –---- 2 –---- 3 -----– 4 ------ 5 –---- 6 –---- 7 –---- 8 ------ 9

26) (Belastbarkeit) Zeigt während oder nach grösseren Anstrengungen wie Transporten und Turnieren vermehrt Anzeichen von Unwohlsein (Flehmen, Koliksymptome, vermehrtes Liegen…)?

Immer Nie

1 –---- 2 –---- 3 -----– 4 ------ 5 –---- 6 –---- 7 –---- 8 ------ 9

Questionnaire (French)

Partie 1:

Date: ______________

Questions générales:

Numéro UELN/SVPS: ____

Nom du cheval: ____

Signalement (Race, Sexe, Age, Robe):____

Discipline (p.ex. saut, dressage etc.):____

Nom du propriétaire et du cavalier/de la cavalière: ___________________________________

Numéro du téléphone: ____

Email: ____

Lieu de détention (canton): ____

Origines/Pedigree : P: ___________________**MP:** __________________________________

Depuis quand le cheval vous appartient-il ou depuis quand le connaissez-vous? _______

Tempérament du cheval?

☐ calme, serein ☐ dynamique, mais coopérant ☐ nerveux, pas très coopérant

Détention du cheval:

1) Votre cheval vit-il dans un groupe des chevaux?

☐ Oui ☐ seulement au pré

☐ Non

2) Combien d'heures par jour le cheval est-il dehors (pré, monté, attelé)?

☐ 0-1h ☐ 1-4h ☐ 4-12h ☐ > 12h

Alimentation:

3) Votre cheval, se bat-il pour la nourriture?

☐ Oui ☐ Non

4) Comment est l'appétit de votre cheval ?

☐ très bien, il mange tout

☐ modéré, parfois il mange avec des petites pauses

☐ insuffisant, le cheval mange seulement lentement et pas tout

Entraînement:

5) Combien de fois est-il entraîné par semaine?

☐ < 1 fois ☐ 4-5 fois ☐ 7 fois

☐ 1-3 fois ☐ 6 fois

6) Combien de personnes montent/entraînent le cheval?

☐ 1 ☐ 2 ☐ 3 ☐ >3

7) Combien de fois par année va-t-il en concours?

☐ 1-3 fois ☐ 4-8 fois ☐ 9-15 fois ☐ 16-25 fois ☐ plus de 15 fois

8) Quelle est le niveau de concours de votre cheval? ________________

9) Comment est la performance du cheval par rapport au niveau de formation?

☐ très bien/bien

☐ suffisante

☐ insuffisante/mauvaise

10) Est-ce que le cheval est motivé durant le travail?

☐ très bien/bien

☐ suffisante

☐ insuffisante/mauvaise

11) Est-ce que la motivation de votre cheval change au cours du travail? (plusieurs réponses possibles)?

☐ Non

Si oui:
☐ au début pas bien
☐ au milieu du travail pas bien
☐ à la fin du travail pas bien
☐ toujours pas bien
☐ changeant
☐ pendant la saison pas bien

12) Est-ce que le cheval a du plaisir naturel de bouger (quand on monte à cheval, sur le pré etc.)?

☐ Oui, beaucoup de plaisir de bouger

☐ Suffisante de plaisir de bouger

☐ Oui, quand on le monte à cheval, mais pas sur le pré

☐ Oui, sur le pré, mais pas quand on le monte à cheval

☐ No, il n’a pas du plaisir de bouger

Stéréotypies:

- Tic à l'ours?	☐ pas du tout	☐ de temps en temps	☐ régulièrement	☐ permanent, excessif
- Tic à l'appui?	☐ pas du tout	☐ de temps en temps	☐ régulièrement	☐ permanent, excessif
- Tic à l'air?	☐ pas du tout	☐ de temps en temps	☐ régulièrement	☐ permanent, excessif
- Tourner en rond?	☐ pas du tout	☐ de temps en temps	☐ régulièrement	☐ permanent, excessif
- Râper les dents contre le bois?	☐ pas du tout	☐ de temps en temps	☐ régulièrement	☐ permanent, excessif

Problèmes de comportement:

- Coller à d'autres chevaux?	☐ pas du tout	☐ de temps en temps	☐ régulièrement	☐ permanent, excessif
- Mordre?	☐ pas du tout	☐ de temps en temps	☐ régulièrement	☐ permanent, excessif
- Taper?	☐ pas du tout	☐ de temps en temps	☐ régulièrement	☐ permanent, excessif
- Ruer?	☐ pas du tout	☐ de temps en temps	☐ régulièrement	☐ permanent, excessif
- Pointer?	☐ pas du tout	☐ de temps en temps	☐ régulièrement	☐ permanent, excessif
- Taper contre la paroi?	☐ pas du tout	☐ de temps en temps	☐ régulièrement	☐ permanent, excessif

Pas du tout	= Aucun signe de stéréotypies ou problèmes comportementaux
De temps en temps	= Stéréotypies ou problèmes comportementaux n'apparaissant pas chaque jour
Régulièrement	= Stéréotypies ou problèmes comportementaux apparaissant chaque jour et se rapportant à un stimulus spécial (par exemple après la nourriture, etc.)
Permanent, excessivement	= Stéréotypies ou problèmes comportementaux apparaissant toute la journée, sauf pendant que le cheval est monté, etc.

Symptômes:

Est-ce que vous avez observé un ou plusieurs des symptômes suivants?

☐ bâillement excessif
☐ le cheval a maigri
☐ le cheval a des problèmes pour prendre du poids
☐ grincement de dents

Partie 2:

(Marquer la bonne valeur d'une croix)

1) (Nervosité) Devient nerveux quand il y a des insectes ou du bruit etc.:

nerveux calme

1 –---- 2 –---- 3 -----– 4 ------ 5 –---- 6 –---- 7 –---- 8 ------ 9

2) (Concentration) Le cheval est facile à entraîner, il n'est pas rapidement distrait et/ou n'est pas facilement dérangé/déconcentré

difficile facile

1 –---- 2 –---- 3 -----– 4 ------ 5 –---- 6 –---- 7 –---- 8 ------ 9

3) (Confiance en soi) Le cheval est-il serein lorsqu'il est seul, séparé du groupe :

excité serein

1 –---- 2 –---- 3 -----– 4 ------ 5 –---- 6 –---- 7 –---- 8 ------ 9

4) (Entraînement) Le cheval est-il facile à entraîner et comprend-il rapidement les exercices :

difficile facile

1 –---- 2 –---- 3 -----– 4 ------ 5 –---- 6 –---- 7 –---- 8 ------ 9

5) (Excitabilité) Est-il rapidement excitable:

Excitable facilement n'est pas excitable

1 –---- 2 –---- 3 -----– 4 ------ 5 –---- 6 –---- 7 –---- 8 ------ 9

6) (l'amabilité envers les hommes) N'est pas agressive ou anxieux:

revêche aimable/affable

1 –---- 2 –---- 3 -----– 4 ------ 5 –---- 6 –---- 7 –---- 8 ------ 9

7) (Curiosité) S'intéresse vivement aux nouveaux objets et s'en approche:

rarement fréquemment

1 –---- 2 –---- 3 -----– 4 ------ 5 –---- 6 –---- 7 –---- 8 ------ 9

8) (Mémoire) Se souvient-il de ce qu'il a appris ou entraîné:

Non excellent

1 –---- 2 –---- 3 -----– 4 ------ 5 –---- 6 –---- 7 –---- 8 ------ 9

9) (Panique) S'affole vite:

fréquemment jamais

1 –---- 2 –---- 3 -----– 4 ------ 5 –---- 6 –---- 7 –---- 8 ------ 9

10) (Coopération) Est coopératif au maniement/soins:

non oui

1 –---- 2 –---- 3 -----– 4 ------ 5 –---- 6 –---- 7 –---- 8 ------ 9

11) (Humeur changeante) Est capricieux/incalculable:

inconstant constant

1 –---- 2 –---- 3 -----– 4 ------ 5 –---- 6 –---- 7 –---- 8 ------ 9

12) (Entêtement) Est entêté quand il refuse un commandement:

entêté s'adapte

1 –---- 2 –---- 3 -----– 4 ------ 5 –---- 6 –---- 7 –---- 8 ------ 9

13) (Docilité) Est en général docile:

insoumis docile

1 ----- 2 ----- 3 ----- 4 ------ 5 ----- 6 ----- 7 ----- 8 ------ 9

14) (Vigilance) Est vigilant vis-à-vis de l'environnement:

jamais toujours

1 ----- 2 ----- 3 ----- 4 ------ 5 ----- 6 ----- 7 ----- 8 ------ 9

15) (Persévérance) Est patient avec nombreux stimuli:

impatient patient

1 ----- 2 ----- 3 ----- 4 ------ 5 ----- 6 ----- 7 ----- 8 ------ 9

16) (l'affabilité envers autres chevaux) Interagit d'une manière amicale avec autres chevaux:

revêche aimable/affable

1 ----- 2 ----- 3 ----- 4 ------ 5 ----- 6 ----- 7 ----- 8 ------ 9

17) (Comportement de concurrence) Est dominant dans les conflits:

dominé dominant

1 ----- 2 ----- 3 ----- 4 ------ 5 ----- 6 ----- 7 ----- 8 ------ 9

18) (Comportement ombrageux) Facile à surprendre:

peureux courageux

1 ----- 2 ----- 3 ----- 4 ------ 5 ----- 6 ----- 7 ----- 8 ------ 9

19) (Timidité) Est timide dans un nouvel environnement:

timide audacieux

1 ----- 2 ----- 3 ----- 4 ------ 5 ----- 6 ----- 7 ----- 8 ------ 9

20) (Coopération) Est coopératif quand on monte à cheval:

jamais toujours

1 ----- 2 ----- 3 ----- 4 ------ 5 ----- 6 ----- 7 ----- 8 ------ 9

21) (Coopération) Est coopératif quand on le charge dans van:

jamais toujours

1 ----- 2 ----- 3 ----- 4 ------ 5 ----- 6 ----- 7 ----- 8 ------ 9

22) (Coopération) Est calme pendant le transport:

jamais toujours

1 ----- 2 ----- 3 ----- 4 ------ 5 ----- 6 ----- 7 ----- 8 ------ 9

23) (Résistance au stress) A besoin de beaucoup de temps pour se reposer après un grand effort tel qu'un concours ou un transport:

Se repose très lentement se repose vite

1 –---- 2 –---- 3 -----– 4 ------ 5 –---- 6 –---- 7 –---- 8 ------ 9

24) (Résistance au stress) Est plus sensible pour des infections après un grand effort tel qu'un concours ou un transport. Par exemple au niveau des voies respiratoires (fièvre, toux, écoulement nasal, conjonctivite etc.):

Très sensible Pas sensible

1 –---- 2 –---- 3 -----– 4 ------ 5 –---- 6 –---- 7 –---- 8 ------ 9

25) (Résistance au stress) Le cheval donne souvent l'impression qu'il ne se sent pas bien après ou durant un grand effort tel qu'un concours ou un transport (par exemple: le cheval baille et se couche souvent, montre des symptômes de colique…)

toujours jamais

1 –---- 2 –---- 3 -----– 4 ------ 5 –---- 6 –---- 7 –---- 8 ------ 9

26) (Résistance au stress) Le cheval n'est pas intéressé par la nourriture après ou durant un grand effort tel qu'un concours ou un transport

toujours jamais

1 –---- 2 –---- 3 -----– 4 ------ 5 –---- 6 –---- 7 –---- 8 ------ 9
